# An exploratory study on the quality of patient screening and counseling for hypertension management in Tanzania

**DOI:** 10.1371/journal.pone.0227439

**Published:** 2020-01-16

**Authors:** Anbrasi Edward, Lisa Hoffmann, Frank Manase, Kunihiro Matsushita, George William Pariyo, Tammy M. Brady, Lawrence J. Appel

**Affiliations:** 1 Department of International Health, Johns Hopkins Bloomberg School of Public Health, Baltimore, MD, United States of America; 2 Patient Safety and Risk Management Unit, World Health Organization, Geneva, Switzerland; 3 Community Center for Preventive Medicine, Dar es salaam, Tanzania; 4 Department of Epidemiology, Johns Hopkins Bloomberg School of Public Health, Division of Cardiology, Johns Hopkins School of Medicine, Baltimore, MD, United States of America; 5 Division of Pediatric Nephrology, Johns Hopkins School of Medicine, Baltimore, MD, United States of America; 6 Departments of Epidemiology and International Health, Johns Hopkins Bloomberg School of Public Health, Johns Hopkins School of Medicine, and Johns Hopkins School of Nursing, Baltimore, MD, United States of America; International University of Health and Welfare, School of Medicine, JAPAN

## Abstract

**Background:**

The global burden of hypertension, currently estimated at 1 billion, is a leading Non-Communicable Disease (NCD) in Sub Saharan Africa. In Tanzania, the reported prevalence of hypertension is 25%. Inherent limitations of the healthcare system to control hypertension include inadequate provider knowledge, system capacity, medication access, and patient awareness, all of which hinder effective screening and disease management. To assess the quality of hypertension screening and patient counseling, we conducted a study in an ambulatory setting in Tanzania.

**Methods:**

Observations of patient screening were conducted on 69 adult patients during routine outpatient care and screening camps. In addition, 33 healthcare providers participated in a pre-post knowledge assessment after observing instructional training videos.

**Results:**

Patient observations indicated that blood pressure (BP) measurement was explained to 65% of patients, and 77% of the measurements were made with mercury sphygmomanometers. For several aspects of BP measurement, nurses performed better than doctors: patient’s arm supported on a flat surface (doctors, 58% vs nurses 67%, p<0.05), and patient’s back was supported (doctors, 50% vs nurses 88%, p<0.01). Among those diagnosed with hypertension, 7% were prescribed medications, 14% were advised on reduced salt during cooking, 29% on reduced salt consumption, 21% on reduced consumption of sodium rich foods, 21% on reducing caloric intake, 21% on increasing physical activity, and 43% were informed about follow up appointments. Provider knowledge assessments showed critical gaps in consequences of hypertension, 1^st^ line medicines, and awareness of guidelines at baseline. Following the instructional videos there were improvements in some aspects: diagnostic criteria for hypertension (pre 45% vs post 91%, p<0.001) and counseling for controlling hypertension (pre 30% vs post 58%, p<0.01).

**Conclusion:**

Enhancing knowledge and performance competencies of health providers at the primary care level is a critical prerequisite for effective hypertension management in low resource settings.

## Introduction

An estimated 36 million die annually due to non-communicable diseases (NCDs), the second leading cause of death globally [1] By 2030, hypertension and other NCDs are projected to exceed communicable diseases as the leading cause of mortality [2]. It has been estimated that 45% of ischemic heart disease deaths and 51% of stroke deaths can be attributed to hypertension [3]. Low and Middle Income (LMIC) countries have a disproportionate burden of cardiovascular disease (CVD) and experience ~80% of global CVD mortality [4]. Regardless of age, the risk of dying from elevated BP in LMICs is more than double that in high-income countries [3]. Seven percent of these deaths occur in adults aged less than 60 years, with higher estimates (25%), reported for the World Health Organization’s African region [3]. According to the World Economic Forum, NCDs associated with urbanization and westernization of diet, lack of exercise, and obesity [5], pose a serious threat to global economic development due to long-term treatment costs and detrimental impacts on productivity [4,6].

Similar to other sub-Saharan countries, hypertension is the most common NCD in Tanzania [7], and impacts approximately 25% of the population [8]. Geographic prevalence estimates vary within the country, ranging from 19% in rural areas to 35% in urban areas with the highest prevalence of 70% in individuals aged 70 and above [6]. The government of Tanzania through the 10 year National Non Communicable Disease Strategy made considerable investments to enhance its capacity to address NCDs [7,9]. However, due to extreme resource constraints and limitations of the health care system, goals for optimal capacity for care were not achieved [7]. Capacity and preparedness of facilities remains an issue, especially in the context of primary care and outpatient management of hypertension [7].

Poor rates of outpatient diagnosis and treatment contribute to the low rates of hypertension control [5]. Other systemic factors include lack of knowledge and skill competencies of health care providers, and availability of blood pressure monitors, laboratory facilities, and medications. The health system is also faced with chronic health workforce deficits with a density of just 0.022 physicians per 1,000 [10] and 0.416 nurses per 1,000 [11]. Lack of awareness and treatment for hypertension have been reported for both healthcare providers and patients [6,12].

In a study from northwest Tanzania of 335 healthcare providers (34 doctors, 68 non-medical clinicians, 150 nurses, and 83 assistants), only 59% showed a fair knowledge of hypertension. Few reported feeling “very comfortable” in management of hypertension, and most facilities were not providing NCD services based on national policies [13]. These facilities routinely refer patients diagnosed with hypertension to hospitals, and counseling of patients has been low [13]. In a comparison of four African countries, Tanzania ranked among the highest in terms of percentage of population reportedly “unaware” of their hypertension, 82% of the 1,790 study participants [4].

A recent national survey of primary health facilities in Tanzania showed that of the 725 facilities, only 42% had guidelines available for hypertension, 9% had a provider who was trained in hypertension, and only 5% had essential equipment available for hypertension services [7]. It is apparent that healthcare providers are not receiving adequate training for hypertension screening and management.

Accurate BP measurement is one critical aspect of effective screening and management of hypertension. Errors in estimating BP can result from several aspects of BP measurement. These include patient talking or fidgeting, patient’s back not supported, small cuff size for patient’s arm, cuff placed over clothing, cuff placed lower or higher than heart level, patients’ legs crossed during measurement, patient’s feet not supported, patient with a full bladder, and patient not rested before the measurement. It has been estimated that these errors in lack of appropriate screening procedures could lead to errors in BP readings of 5 to 40mmHg [14].

In this context, we conducted an exploratory study to determine the quality of patient screening and counseling for hypertension and test whether instructional training videos at an outpatient clinic and screening camp in Tanzania can improve knowledge among health care providers.

## Methods

This study was conducted at Community Center for Preventive Medicine (CCPmedicine), in Dar-es-salaam in Tanzania. To address the growing disease burden of NCDs, efforts were made by the CCPmedicine to develop a comprehensive health service delivery model providing health services to a catchment area population of 276,457, and hypertension screening at schools, churches, mosques, workplaces, supermarkets, marketplace etc. The model institutes multi-disciplinary strategies integrating facility and community mechanisms to enhance awareness, screening and management of NCDs, particularly hypertension. A cadre of clinicians and nurses provide routine outpatient care in the clinic, and hypertension screening in health camps. The center has received recognition from national and sub-national policy makers, and leaders including the recent 2018 World Health Forum in Geneva. Deliberations with the director of CCPmedicine indicated that over 200 physicians and nurses from the private and public sector participate in the center’s activities for hypertension screening and management, few of which have received training specifically for hypertension screening, and no evaluations have been conducted on provider knowledge, competencies and performance.

Sixty-nine patient consultations conducted by a total of 20 health providers were observed by a United States trained nurse over a period of two weeks. A consecutive sample of all new adult patients, 30 years and older who presented at the clinic and at a camp were included in the sample. Patients who presented for follow up visits or required urgent referral were excluded. All providers who were engaged in patient screening and management were included in the clinical assessments. No reassessments were performed to validate blood pressure readings.

We also pilot tested instructional training videos which were developed by a team at Johns Hopkins University (https://www.youtube.com/watch?v=3EMcIVWSmPk, https://www.youtube.com/watch?v=T9J3RE4Eins) to improve hypertension knowledge, screening and management in LMIC. All health providers including doctors, nurses, and other health providers who screen and manage patients were included in the pre-post knowledge assessments. The pre-post tests were administered to 33 health providers who observed the instructional videos. The post tests were conducted immediately following the screening of the instructional videos. A facility audit was also administered with the supervisor of the clinic to determine facility capacity for trained providers, availability of BP devices, medications etc.

Validated instruments employed by other studies on hypertension were modified for the provider observations, interviews, and instructional video testing. These included the Johns Hopkins Rich Life Project Assessment Tool, Blood Pressure Measurement Fidelity Audit Report, CDC Measure Up Pressure Down Medical Assistant Training Performance Checklist, CDC Measure Up Pressure Down Medical Assistant Training Written Test, and WHO Assessment Tool for Prevention and Control of Noncommunicable Diseases: Guidelines for Primary Health Care in Low Resource Settings. Data was entered in Epi Info Version 7.2 for Windows, cleaned and analyzed using STATA 14. Descriptive statistics were performed and paired and unpaired t tests were used to assess differences by provider cadre and pre-post knowledge competency test scores, for variables with adequate sample size. Written consent for participation was obtained from all health providers who were interviewed or observed, and from the facility supervisor. This included purpose and objectives of the study, risks, discomforts and benefits, confidentiality and contact information for concerns regarding the study.

The study was considered Human Subjects Exempt by the Johns Hopkins Bloomberg School of Public Health Institutional Review Board since we did not collect any data from patients. CCPmedicine had ethical clearance for conducting assessments on hypertension prevention and management from the National Institute for Medical Research in Tanzania.

## Results

A total of 69 patient observations were conducted in the clinic and hypertension camp. Table 1 provides characteristics of providers who were observed and those that participated in the instructional video testing. Not all providers who participated in the video testing had patient care interactions during our observation. A majority of the observed providers were male (60%), between the ages of 20–30 years, and 60% of the sampled providers were nurses. Sixty-five percent of these reported more than three years of experience. The profiles of providers who participated in the knowledge and skill competency assessment and instructional video testing were similar to those who were observed, but they also included 4 providers who were lab technicians, a radiographer and receptionist, who also perform screening of outpatients.

**Table 1:** Selected Characteristics of Sampled Providers for a) Clinical Observations and b) Video Testing.

Provider Characteristics	Providers		Video Testing	
	N = 20	%	N = 33	%
Provider Gender				
*Male*	12	60	13	39
*Female*	8	40	20	61
Provider Age				
*20-30y*	14	70	24	73
*31-45y*	6	30	9	27
Provider Cadre				
*Doctor*	8	40	8	24
*Nurse*	12	60	21	64
*Lab technician*, *Radiographer*, *Receptionist*	-	-	4	12
Provider Experience				
*<3y*	7	35	10	30
*3-5y*	8	40	14	43
*6–10*	3	15	5	15
*>10y*	2	10	4	12

Table 2 documents adherence to screening standards. A majority of the patients were screened with a mercury sphygmomanometer (77%). Adherence to standards for patient preparation prior to BP measurements based on clinical observations showed that about 65% of the patients were informed by the providers that their blood pressure was being measured, 70% had rested prior to the measurement, 74% had their back supported, all patients had their feet supported, 91% had their feet uncrossed, 64% had their arm supported on a flat surface, and 80% ensured that the patient did not speak, or text or talk on their cell phone. There was a significantly higher adherence by nurses than by doctors for ensuring that the patient’s back was supported (50% vs 88%, p = 0.003) and their arm was rested on a flat surface (58% vs 67%, p = 0.041).

**Table 2:** Screening of adult patients for hypertension by provider cadre.

Screening Tasks	Doctors	Nurses	Total
	**N = 26**	**N = 43**	**N = 69**
	n(%)	n(%)	n(%)
Type of BP Screening Device			
*Automated device*	4 (15)	12 (28)	16 (23)
*Mercury device*	22 (85)	31 (72)	53 (77)
Patient Preparation (automated and manual)			
*Explained to patient that BP is going to be measured*	19 (73)	26 (60)	45 (65)
*Ensured patient rested 2–5 minutes before measurement*	21 (80)	27 (63)	48 (70)
*Ensured patient back was supported*	13 (50)	38 (88)*	51 (74)
*Ensured patient feet were supported*	26 (100)	43 (100)	69 (100)
*Ensured patient feet and legs are uncrossed*	23 (88)	40 (93)	63 (91)
*Ensured arm is rested on flat surface*	15 (58)	29 (67) *	44 (64)
*Ensured patient does not speak*, *text*, *or use cell phone during measurement*	23 (88)	32 (74)	55 (80)
Measurement of Blood Pressure (**Automatic device**)	**N = 4**	**N = 12**	**N = 16**
*Chose correct cuff size by measuring arm circumference*^*1*^	N/A	N/A	N/A
*Placed cuff on bare arm*	3 (75)	9 (75)	12 (75)
*Aligned middle of cuff (denoted by cuff marking) with brachial artery*	None	2 (17)	2 (13)
*Placed cuff at mid-heart level*	4 (100)	10 (83)	14 (88)
*Took a 30 second rest period between subsequent measurements*^*2*^	None	None	None
*Repeated reading on opposite arm if previous reading was abnormal*^*3*^	None	None	None
*Documented the confirmatory measurement is average of first 3 measurements*^*4*^	None	None	None
Measurement of Blood Pressure (**Manual device**)	**N = 22**	**N = 31**	**N = 53**
*Tightly closed BP cuff valve stem*	22 (100)	31 (100)	53 (100)
*Placed stethoscope ear pieces secure in ears in correct direction*	22 (100)	31 (100)	53 (100)
*Took a 30 second rest period between subsequent measurements n = 12 for doctors and n = 8 for nurses*	8 (67)	1 (13)	9 (45)
*Repeated reading on opposite arm*, *if previous reading was abnormal*	2/5 (40)	None	2/13 (15)
*Documented the confirmatory measurement was the average of the first 3 measurements taken*	None	None	None

* p<0.05

^*1*^NA only 1 cuff was available

^2,3,4^Only 2 patients had 2 readings taken, but providers did not wait for 30 seconds or perform the measure with the opposite arm, nor averaged the readings

Note t tests were not performed for all indicators due to small sample size

For 75% of patients, the providers ensured that the cuff was placed on the bare arm, 88% placed at mid-heart level, but only 13% had the middle of the cuff (denoted by cuff marking) aligned with the brachial artery. A second BP reading was obtained only for 2 patients, but the providers did not ensure a 30 second rest between the readings. For patients whose BP was measured with a manual device, most providers adhered to the measurement standards; all tightly closed the valve system, all placed the stethoscope ear pieces correctly. Only 45% of the patients had a 2^nd^ reading, but 45% of providers ensured that they had a 30 second rest before the 2^nd^ measure. Of those who had a previously abnormal reading, only 13% had the 2^nd^ measure in the opposite arm.

Of the 14 patients who were diagnosed with hypertension, based on elevated blood pressure readings, only 21% had confirmatory lab tests ordered, and only 1 patient was prescribed medication, but the provider explained the regimen, potential side effects, importance of compliance to medication and where to obtain the medication (Table 3). Diet and lifestyle counseling of patients who were diagnosed with hypertension was also low; 14% were advised about reducing salt in cooking, 29% reduced table salt consumption, 21%, reducing consumption of sodium rich foods, caloric intake, and increasing physical activity, and 43% were informed on follow up assessments. Only 1 patient who was a smoker was counseled about quitting smoking and reducing alcohol consumption.

**Table 3:** Management of patients diagnosed with hypertension.

Management Tasks for Hypertension	N = 14
	n (%)
Order other lab tests	3 (21)
Explain the lab tests	2/3 (67)
Prescribe blood pressure medications	1 (7)
Type of Medicines Prescribed	
*Furosemide*	1 (7)
Patient Counseling	
*Explained medication regimen*	1 (100)
*Explained potential side effects of medication*	1 (100)
*Explained the importance of taking the medication*	1 (100)
*Explained where to obtain the medication*	1 (100)
*Reducing salt in cooking*	2 (14)
*Reducing table salt consumption*	4 (29)
*Reducing consumption of foods rich in sodium*	3 (21)
*Reducing caloric intake*	3 (21)
*Increasing physical activity or exercise*	3 (21)
*Inform about follow up appointments*	6 (43)

Provider knowledge competency assessments were conducted prior to the viewing of the two instructional videos which provided general information on the global burden and consequences of hypertension and on patient preparation prior to BP measurement (Table 4). A post-test was also conducted following the video training to determine improvements in knowledge. At pre-test, all doctors and 92% of the nurses strongly agreed or agreed that they had adequate knowledge about hypertension disease and adequate skills for blood pressure measurement. However, there was a significant improvement in the knowledge of thresholds used to diagnose hypertension following the video training (45% vs 91%, p<0.001). Notably, knowledge on all the potential consequences of hypertension (heart attack, stroke, death, kidney failure), did not improve, as some providers only indicated, heart attack, stroke or death. There was a significant improvement in knowledge of critical factors to control blood pressure such as maintaining healthy lifestyle, adhering to medications, and following up regularly with health providers, following the video screening (30% vs 58%, p<0.001). Knowledge of lifestyle changes to control hypertension did not change significantly (82% vs 91%). Likewise, provider awareness that hypertension management is lifelong did not improve significantly (64% to 76%). However, there was a significant improvement in post-test results in awareness that patients with hypertension often show no symptoms (33% vs 58%, p<0.01).

**Table 4:** Provider knowledge and competency assessment of hypertension by provider cadre.

Knowledge Components	Doctors (n = 8)		Nurses & Other (n = 25)		Total (n = 33)	
	Pre	Post	Pre	Post	Pre	Post
Providers Strongly Agree/Agree:	n(%)	n(%)	n(%)	n(%)	n(%)	n(%)
*Adequate knowledge for blood pressure measurement*	8 (100)	8 (100)	23 (92)	24 (96)	31 (94)	32 (97)
*Adequate skills for blood pressure measurement*	8 (100)	8 (100)	23 (92)	25 (100)	31 (94)	33 (100)
Cut off for Diagnosis of hypertension						
*120/90*	1 (13)	-	3 (12)	-	4 (12)	-
*130/80*	1 (13)	-	1 (4)	1 (4)	2 (6)	1 (3)
*150/90*	2 (25)	-	10 (40)	2 (8)	12 (36)	2 (6)
*140/90*	4 (50)	8 (100)	11 (44)	22 (88)	15 (45)	30 (91)***
*135/70*	-	-	-	-	-	-
Knowledge on consequences of Hypertension						
*Heart attack*	-	1 (13)	3 (12)	2 (8)	3 (9)	3 (9)
*Stroke*	2 (25)	1 (13)	2 (8)	3 (12)	4 (12)	4 (12)
*Death*	1 (13)	1 (13)	3 (12)	5 (20)	4 (12)	6 (18)
*Kidney failure*	-	-	-	-	-	-
*All the above*	5 (63)	5 (63)	17 (68)	15 (60)	22 (67)	20 (61)
Aware that systolic blood pressure is the first sound heard	7 (88)	7 (88)	14 (56)	15 (60)	21 (64)	22 (67)
Aware of critical factors for controlling blood pressure						
*Maintaining healthy life style*	3 (38)	1 (13)	9 (36)	2 (8)	12 (36)	3 (9)
*Taking medications*	-	-	-	2 (8)	-	2 (6)
*Following up regularly with health* *providers*	1 (13)	1 (13)	10 (40)	8 (32)	11 (33)	9 (27)
*All the above*	4 (50)	6 (75)	6 (24)	13 (52)	10 (30)	19 (58)**
Lifestyle changes to control Hypertension						
*Reducing amount of salt in diet*	1 (13)	1 (13)	1 (4)	-	2 (6)	1 (3)
*Eating healthy foods*	-	-	1 (4)	-	1 (3)	-
*Quitting smoking*	-	-	-	-	-	-
*Losing weight*	-	-	-	-	-	-
*Exercising regularly*	-	-	3 (12)	2 (8)	3 (9)	2 (6)
*Limiting alcohol*	-	-	-	-	-	-
*All the above*	7 (88)	7 (88)	20 (80)	23 (92)	27 (82)	30 (91)
Knowledge Awareness:						
*Hypertension management is lifelong*	6 (75)	7 (88)	15 (60)	18 (72)	21 (64)	25 (76)
*Blood pressure is the force being applied against arterial walls as the heart pumps blood throughout the body*	8 (100)	8 (100)	21 (84)	21 (84)	29 (88)	29 (88)
*Hypertension often shows no symptoms*	4 (50)	6 (75)	7 (28)	13 (52)	11 (33)	19 (58)*
*Correct definition of Myocardial Infarction*	6 (75)	5 (63)	5 (20)	2 (8)	11 (33)	7 (21)
*Healthy diets can reduce systolic blood pressure by as much as 10 mmHg*	5 (63)	5 (63)	16 (64)	9 (36)	21 (64)	14 (42)
*Errors in blood pressure reading*	4 (50)	5 (63)	12 (48)	16 (64)	16 (48)	21 (64)
*Blood pressure deflation rate*	5 (63)	5 (63)	11 (44)	17 (68)	16 (50)	22 (67)
*Brachial artery is used for measuring blood pressure*	6 (75)	7 (88)	11 (44)	13 (52)	17 (52)	20 (61)
*Correct cuff application to the arm is determined by placing 2 fingers under the bottom edge of the cuff*	6 (75)	7 (88)	14 (56)	19 (76)	20 (61)	26 (79)
*Timing between blood pressure readings*	8 (100)	5 (63)	14 (56)	18 (72)	22 (67)	23 (70)
*Standard Treatment Guidelines for Hypertension*	7 (88)	-	12 (48)	-	19 (58)	-
*Part of the stethoscope used to measure blood pressure*	7 (88)	6 (75)	21 (84)	21 (84)	28 (85)	27 (82)
*Ministry standards for number of blood pressure readings at first visit to confirm hypertension*	5 (63)	NA	18 (72)	NA	23 (70)	NA
*Importance of ensuring patient did not smoke*, *exercise*, *or drink a caffeinated beverage for at least 30 minutes before measurement*	6 (75)	7 (88)	21 (84)	22 (88)	27 (82)	29 (88)
*Correct placement of blood pressure cuff*	7 (88)	6 (75)	14 (56)	12 (48)	21 (64)	18 (55)
*Blood pressure threshold for patients with diabetes*, *cardiac or renal impairment*	1 (13)	NA	3 (12)	NA	4 (12)	NA
*National guidelines for maximum sodium intake*	7 (88)	NA	15 (60)	NA	22 (67)	NA
*Protocol for follow up of hypertensive patients*	6 (75)	NA	20 (83)	NA	26 (81)	NA
Awareness of First line HT medicines based on national protocol						
*Thiazides*	5 (63)	NA	6 (24)	NA	11 (33)	NA
*Beta-Blockers*	5 (63)	NA	2 (8)	NA	7 (21)	NA
*Calcium Channel Blockers*	6 (75)	NA	5 (20)	NA	11 (33)	NA
*ACE Inhibitors*	4 (50)	NA	3 (12)	NA	7 (21)	NA
*Blockers (ARBs)*	2 (25)	NA	9 (36)	NA	11 (33)	NA

*p<0.05

** p<0.01

***p<0.001

Note t tests were not performed for all indicators due to small sample size

Though there were some improvements in knowledge, most changes in knowledge were not statistically significant. These included errors in blood pressure reading, blood pressure deflation rate, brachial artery use for blood pressure readings, determining cuff size, timing between blood pressure readings, awareness of national standards, and taking precautions for patient preparation 30 minutes prior to blood pressure readings (patient does not smoke, exercise, drink caffeine). However, the instructional videos only focused on certain knowledge and patient preparation elements, and other aspects were shared following the pre-testing. There was an unanticipated reduction in knowledge scores related to the definition of myocardial infarction following the video (33% vs 21%, p<0.0436). Very few were aware of blood pressure threshold for patients with diabetes and cardiac or renal impairment, though a higher proportion of providers were aware of the national guidelines for sodium intake; >80% were aware of the protocols for follow up of hypertensive patients.

There was either strong agreement or agreement on the value and functionality elements of the instructional videos (Table 5). More than 75% of the providers reported that the videos were appropriate for patient age, gender, and ethnicity. Over 85% reported that the introduction was motivating and stimulated interest, provided clear objectives, simplified complex tasks, allowed learners to reflect on clinical practice, repeated learning elements at the conclusion of the video, was conducive to learner interaction, and had appropriate vocabulary level and speed of narration.

**Table 5:** Provider assessment of video functionality and value assessment (agree or strongly agree).

Functionality Characteristics	
	N = 33
	n(%)
Accuracy of Video content	33 (100)
Intent to incorporate ideas presented in the video in clinical practice	31 (94)
Appropriate for age	27 (82)
Appropriate for sex	28 (85)
Appropriate for ethnicity	29 (88)
Appropriate for physically impaired	26 (79)
Appropriate for local values	30 (91)
Appropriate dress code	30 (91)
Appropriate for language	26 (79)
Appropriate for social class	30 (91)
Introduction was motivating to stimulate interest	33 (100)
Objectives and key elements were clear	33 (100)
Simplified complex tasks and avoided introducing unnecessary or irrelevant information	32 (97)
Suggested methods for me to apply the newly acquired knowledge	33 (100)
Allowed to reflect on clinical practice during the viewing	32 (97)
Illustrations in video aided learning	32 (97)
Learning elements were repeated in the conclusion of the video	32 (97)
Conducive to learner interaction	29 (88)
Well organized and structured	31 (94)
The visual quality did not detract from the overall message and content	30 (91)
The vocabulary of the narration was appropriate for the intended audience	32 (97)
The speed of the narration was slow enough to be understood	31 (94)
Sound effects added emphasize on certain aspects and enhanced learning	32 (97)
The terms were well defined	32 (97)
The references of the content were included	25 (76)

A structured interview with the clinic supervisor indicated that the clinic had 984 patients with hypertension who were registered and followed up. Though providers received refresher training in the past year, there were no clinical guidelines, treatment algorithms, or any instructional educational materials available for providers. However, some brochures were available for patients. Through its clinic-based services and camp screenings, patients were provided free follow up screening, and counseling on smoking cessation, alcohol reduction, and dietary and lifestyle changes. The clinic provides pharmacy services for purchasing hypertensive medications with fee exemptions for patients with lower incomes. Common patient barriers reported for appropriate hypertension control were lack of patient awareness on importance of follow up visits, lack of facility access, affordability of prescribed medications, dual practice of traditional healers and medicine, inability to reduce salt consumption, and lack of patient awareness on high sodium foods.

## Discussion

The findings showed knowledge gaps in providers for hypertension diagnosis and management. Nurses showed better adherence to clinical standards than doctors for some aspects. A one-time exposure to the video-based instruction improved knowledge for some content that were addressed in the videos. This exploratory study provides useful insights on the knowledge and performance gaps of providers in primary care clinics who routinely screen adult patients in ambulatory care settings and the value of simple instructional videos to provide continued learning, thereby enhancing provider knowledge and performance. The gaps in knowledge ranged from standard clinical procedures for patient screening and management, knowledge about the disease and complications, and appropriate patient counseling.

To enhance hypertension control at the facility level, appropriate training of providers is essential to ensure quality of screening, management, and patient counseling. The videos were primarily created for non-clinicians who may provide hypertension services and patients or their family members who may be interested in hypertension, yet it is evident that even doctors lacked basic knowledge on certain factors. Following the post-test, there was a high demand for additional educational materials and videos for improving knowledge and skills in disease management. Many of the providers who participated in the testing remained in the clinic to learn the correct responses to the test and practice the correct positioning and patient preparation to improve their skills. The staff noted that equipment functionality is a common problem, e.g. cuffs were frayed, and different sizes were unavailable.

The study illustrates key competency gaps in the current systems of service delivery. Other studies on hypertension in Tanzania have focused on prevalence and associated risk factors of hypertension [6]. A study in Magu district, with 9678 participants, reported an 8.2% prevalence of hypertension and 36.2% prevalence of pre-hypertension among adults, with a higher prevalence among older adults and females [6]. Hypertension awareness among adults was only 10%, with significant differences between women (11.1%) and men (5.3%, P < 0.003), and between urban (11.9%) and rural dwellers (7%, p < 0.02). Of the 773 hypertensive individuals, only 7.1% had been treated with antihypertensive drugs, with a somewhat higher frequency (9.2%) in urban areas compared to rural areas (5.3%, p < 0.03), and in females (8.5%) compared to males (4.1%, p < 0.03). The rates are much lower for patients who are poorly controlled, which is postulated to be due to the lack of access to hypertensive drugs, and compliance to medications. The counseling component of this study showed poor performance, indicating the need for enhancing provider capacity to effectively screen, treat and counsel patients. Integrated service delivery was proposed to effectively screen for hypertension in adults attending outpatient services. Our study provides some useful options for improving hypertension knowledge and screening using simple video based instructional resources.

In another study of 139 teaching staff from higher learning institutions in Tanzania, knowledge about the causes, signs and symptoms, risk factors and complications for hypertension was suboptimal. The major source of information for learning about hypertension was the internet and media, highlighting the need for focused training on hypertension management [12]. Another study on 24 public and not for profit health facilities in urban and rural Tanzania also showed that only 59% had fair knowledge of hypertension, and only 33% had seen more than five patients with hypertension in the past three months. Only one hospital reported training for hypertension, and only 5 facilities reported receiving a monitoring or supervision visit in the past three months for hypertension [5]. These findings further highlight the need for capacity investments to improve hypertension screening and management. The national study on Tanzania Service Provision Assessment, conducted between 2014–2015, also showed that only 28% showed facility preparedness, with 42% of facilities having hypertension guidelines and only 9% where at least one staff member had received training in hypertension. Basic drugs for hypertension treatment were also lacking with only 7% of facilities having all the essential medicines, 21% with ACE inhibitors, 37% with thiazide diuretics, and 16% with beta blockers [7]. These systemic deficiencies may have led to poor provider adherence in these studies.

As described earlier, it is apparent that considerable capacity investments are essential to effectively screen for and control hypertension in Tanzania. The knowledge capacity and performance competency of health providers are of paramount importance as a fundamental prerequisite to addressing the disease burden. In recognition of the growing burden of non-communicable diseases, CCPmedicine has launched several health facility and community oriented initiatives to raise awareness, access and engagement of a wider audience of professional, government, school and community providers to address the burden of hypertension in Tanzania.

This study has limitations. This study was designed to examine the current practice and performance of the hypertension services in this private clinic, and therefore may not be representative of other public and private clinics. Second, there is the possibility of observer bias; however, there was no consistent pattern of poor or high quality. Third, the study did not obtain data from patients. Further empirical research on representative samples of public and private primary health facilities in rural and urban settings in Tanzania would provide more generalizable estimates of current practices, clinical performance and training needs of primary care providers who are engaged in the routine screening and management of patients with hypertension. In future studies, we plan to increase the sample of clinics, providers and patients.

## Conclusion

The study has illustrated inherent knowledge and performance gaps in health providers conducting routine screening and management of hypertension in primary care settings. The results from the instructional video testing show the potential for instituting relatively inexpensive measures for improving performance in low resource contexts. However, efforts must include system level investments for appropriate policies, guidelines, commodities, supervisory oversight in addition to improving the knowledge and skill competencies for health providers to effectively prevent and manage hypertension in countries like Tanzania.

## Supporting information

S1 Data TanzaniaFormA3. (XLS)

S2 Data TanzaniaFormB3. (XLS)

## Abbreviations

NCD: Non-communicable diseases
BP: Blood Pressure
